# Preliminary experience with dosimetry, response and patient reported outcome after ^177^Lu-PSMA-617 therapy for metastatic castration-resistant prostate cancer

**DOI:** 10.18632/oncotarget.12240

**Published:** 2016-09-24

**Authors:** Wolfgang P. Fendler, Svenja Reinhardt, Harun Ilhan, Andreas Delker, Guido Böning, Franz J. Gildehaus, Christian Stief, Peter Bartenstein, Christian Gratzke, Sebastian Lehner, Axel Rominger

**Affiliations:** ^1^ Department of Nuclear Medicine, Ludwig-Maximilians-University of Munich, Munich, Germany; ^2^ Department of Molecular and Medical Pharmacology, David Geffen School of Medicine at UCLA, Los Angeles, CA, USA; ^3^ Department of Urology, Ludwig-Maximilians-University of Munich, Munich, Germany; ^4^ Comprehensive Cancer Center, Ludwig-Maximilians-University of Munich, Munich, Germany

**Keywords:** mCRPC, PET, prostate cancer, PSMA, lutetium

## Abstract

Prostate cancer can be targeted by ligands to the prostate-specific membrane antigen (PSMA). We aimed to evaluate dosimetry, safety and efficacy of ^177^Lu-PSMA-617 radioligand therapy (RLT) in patients with metastatic castration-resistant prostate cancer (mCRPC).

Fifteen patients each received two cycles of 3.7 GBq (*n* = 5) or 6.0 GBq (*n* = 10) ^177^Lu-PSMA-617 at an eight to ten weeks interval. For safety monitoring, each treatment was followed by dosimetry with serial quantitative SPECT as well as inpatient and outpatient recording of adverse events. Response to RLT was primarily determined by baseline to follow-up change in ^68^Ga-PSMA PET/CT (RECIST1.1), as well as change in prostate-specific antigen (PSA), quality of life (QoL, FACT-P scale), and pain (Brief Pain Inventory) as secondary endpoints.

Radiation dose delivered to the tumor (6.1 Gy/GBq) was six to twelve-fold higher than to critical organs (kidney left/right 0.5/0.6 Gy/GBq each, salivary glands 1.0 Gy/GBq). Total radiation dose per kidney did not exceed 23 Gy in any patient. Three patients had sub-acute and latent grade 3 events, i.e. anemia, leukocytopenia, and nausea. No acute events, grade ≥4 events or high grade events for salivary gland or kidney function were observed. After two RLT cycles, 4 (27%) patients had partial response, 6 (40%) had stable disease, and 5 (33%) had progressive disease according to RECIST. Any PSA decline was observed in 12/15 (80%) patients during RLT. Significant pain relief was documented in 7/10 (70%) symptomatic patients and QoL improved in 9/15 (60%) patients.

^177^Lu-PSMA-617 therapy proved safe and indicated promising response rates for both objective and patient-reported outcomes in our small group of mCRPC patients.

## INTRODUCTION

After non-melanoma skin tumors, prostate cancer (PCa) is the second most common malignancy in men, and ultimately causes more than 250,000 deaths worldwide each year [1]. Radionuclide therapy has become an effective treatment option for metastatic disease with the approval of bone seeking radiopharmaceuticals; systemic application of the calcium ion mimetic ^223^Ra improves survival in patients with castration-resistant PCa and symptomatic bone metastases [2]. However, about one third of patients with metastatic castration-resistant prostate cancer (mCRPC) present with lymph node or visceral metastases, which are un-responsive to bone-seeking agents [3]. Radiolabeled ligands to the prostate-specific membrane antigen (PSMA) have recently been developed to image and target systemic disease. PSMA is over-expressed in PCa, and this over expression increases further in cases of de-differentiated, metastatic or hormone-refractory disease [4]. The ^177^Lu labeled small molecule ligand DKFZ-PSMA-617 (^177^Lu-PSMA-617) binds with high affinity to PSMA *in vitro* and *in vivo* [5-6]. The potential of ^177^Lu-PSMA-617 for radioligand therapy (RLT) has been explored in several preclinical and clinical studies, among which our previous report on human dosimetry demonstrated favorable body distribution and high tumor-to-organ uptake ratios [6]. Based on our dosimetry calculations, we identified kidney as activity-limiting organ and recommended 6.0 GBq as an appropriate activity for the initial ^177^Lu-PSMA-617 RLT cycle [6], giving high tumor dosing without excessive irradiation of vulnerable healthy organs. Following upon this result, we now aim to provide data on clinical safety and efficacy of ^177^Lu-PSMA-617 RLT in an expanded patient cohort after a total of 30 ^177^Lu-PSMA-617 RLT cycles. Efficacy was based both on objective endpoints and patient-reported metrics, including pain intensity and quality of life (QoL) scores. To complete dose safety evaluation, we further aimed to perform accurate dosimetry of salivary glands, the organ with highest absorbed radiation dose.

## RESULTS

### Characteristics of the study cohort

Baseline clinical and demographic characteristics are given in Table 1. All patients completed two cycles of RLT. Five patients were unfit for chemotherapy prior to RLT. 14 of 15 (93%) patients underwent Abiraterone or Enzalutamide therapy before ^177^Lu-PSMA-617 RLT. One patient did not tolerate second or third line hormonal therapy. Patients had undergone a median of 3 (range, 1 to 5) courses of hormonal therapy prior to admission. Patients with ongoing hormonal therapy at the start of RLT continued their therapy until at least the final follow-up in the present study. None of the patients discontinued gonadotropin-releasing hormone analogs within three months before RLT.

**Table 1:** Baseline characteristics of the patients

Characteristic (*n* = 15)	Median (range) or total number (%)	
Age	73	(54 - 81)
ECOG		
0	5	(33%)
1	5	(33%)
2	5	(33%)
Gleason sum		
7	1	(7%)
8	1	(7%)
9-10	13	(87%)
Sites of metastases		
Bone	14	(93%)
Lymph node	12	(80%)
Liver	3	(20%)
Other	2	(13%)
Lung	1	(7%)
Mean pain intensity score at baseline (0-10)		
0	5	(33%)
1-5	8	(53%)
6-10	2	(13%)
Biochemical values		
Lactate dehydrogenase (U/L)	287	(167 - 1220)
Hemoglobin (g/dL)	12.3	(8.3 - 15.5)
Total alkaline phosphatase (U/L)	147	(49 - 420)
PSA (μg/L)	388	(3.2 - 10661)
No. of prior hormonal therapies		
1	1	(7%)
2	4	(27%)
3	7	(47%)
≥4	3	(20%)
No. of prior chemotherapy regimens		
0	5	(33%)
1	5	(33%)
≥2	5	(33%)
Prior ^223^Ra	5	(33%)
Prior EBRT	9	(60%)

Abbreviations: ECOG, Eastern Cooperative Oncology Group Performance Status; EBRT, External Beam Radiation Therapy.

### Dosimetry

Mean±SD radiation dose by organ calculated from serial quantitative SPECT and blood activity concentrations is given in Table 2. The highest organ radiation dose was observed for salivary glands (1.0±0.6 Gy/GBq) and kidneys (right/left 0.6±0.2/0.5±0.3 Gy/GBq). The mean radiation dose to tumor lesions was 6.1±4.9 Gy/GBq resulting in a mean tumor-to-kidney dose ratio of 11.1 and mean tumor-to-salivary-gland ratio of 6.1. Liver, spleen and bone marrow all received doses ≤ 0.1 Gy/GBq. Maximum cumulative kidney dose (10.3 Gy) and salivary gland dose (16.5 Gy) were below the dose range reported as critical (23 Gy for kidney [7]; 26-50 Gy [8-9] for salivary glands).

**Table 2:** Radiation dose after 30 cycles of

Organ (*n* = 30)	Mean (Gy/GBq)	SD
Dose limiting organs		
Kidney left	0.5	0.3
Kidney right	0.6	0.2
Salivary glands*	1.0	0.6
Non-dose limiting organs		
Liver	0.1	0.1
Spleen	0.1	0.1
Bone marrow	0.002	0.005
Tumor^‡^	6.1	4.9

* determined in ten patients with 2×6.0 GBq ^177^Lu-PSMA-617 RLT, *n* = 20; ^‡^up to three lesions with highest tracer uptake per patient and cycle (*n* = 5 visceral metastases, *n* = 12 LN metastases, *n* = 22 bone metastases).

### Safety

No acute or grade ≥4 adverse events were observed. Sub-acute and latent adverse events occurring in at least one patient are listed in Table 3. Grade 1 and 2 adverse events occurred almost equally often in patients with 3.7 versus 6.0 GBq RLT (3.4 events/patient versus 4.2 events/patient). During inpatient stay, one instance of grade 3 anemia, and during follow-up one case of grade 3 leukocytopenia were noted in patients with 6.0 GBq RLT; both conditions had improved at final follow-up without requiring transfusion or bone marrow stimulation. No event for neutropenia was recorded. At intermediate follow-up, grade 3 nausea was noted in one patient after the second cycle of 3.7 GBq RLT, which responded well to antiemetic medication. Mild or transient xerostomia was reported both for patients with salivary gland dose above (*n* = 4) versus below (*n* = 3) the median. One patient presented with new onset of epigastric pain and markedly elevated gamma-glutamyl transferase at final follow-up. PET/CT demonstrated progression of liver metastases with infiltration of hepatic veins and intrahepatic cholestasis. The patient died six weeks later from acute liver failure associated with progressive tumor infiltration. In total, four patients died from PCa-related events during the observation period, at 24, 28, 36 and 42 weeks after start of RLT.

**Table 3:** Adverse events after 30 cycles of

Adverse event	Latent		Sub-acute	
	All grades	Grade 3	All grades	Grade 3
Renal				
Glomerular filtration rate (GFR)	8 (53%)	0	1 (7%)	0
Tubular extraction rate (TER)	2 (13%)	0	-	-
Hyperkalemia	4 (27%)	0	0	0
Hematologic				
Anemia	3 (20%)	0	9 (60%)	1 (7%)
Thrombocytopenia	2 (13%)	0	1 (7%)	0
Leukocytes	7 (47%)	1 (7%)	1 (7%)	0
Liver				
Bilirubin	0	0	1 (7%)	0
AST/ALT	2 (13%)	0	1 (7%)	0
Other				
Fatigue	5 (33%)	0	0	0
Dry mouth	7 (47%)	0	0	0
Nausea	5 (33%)	1 (7%)	0	0
Dysgeusia	3 (20%)	0	0	0

Absolute number of adverse events (%) are given.

### Efficacy

**Figure 1:**
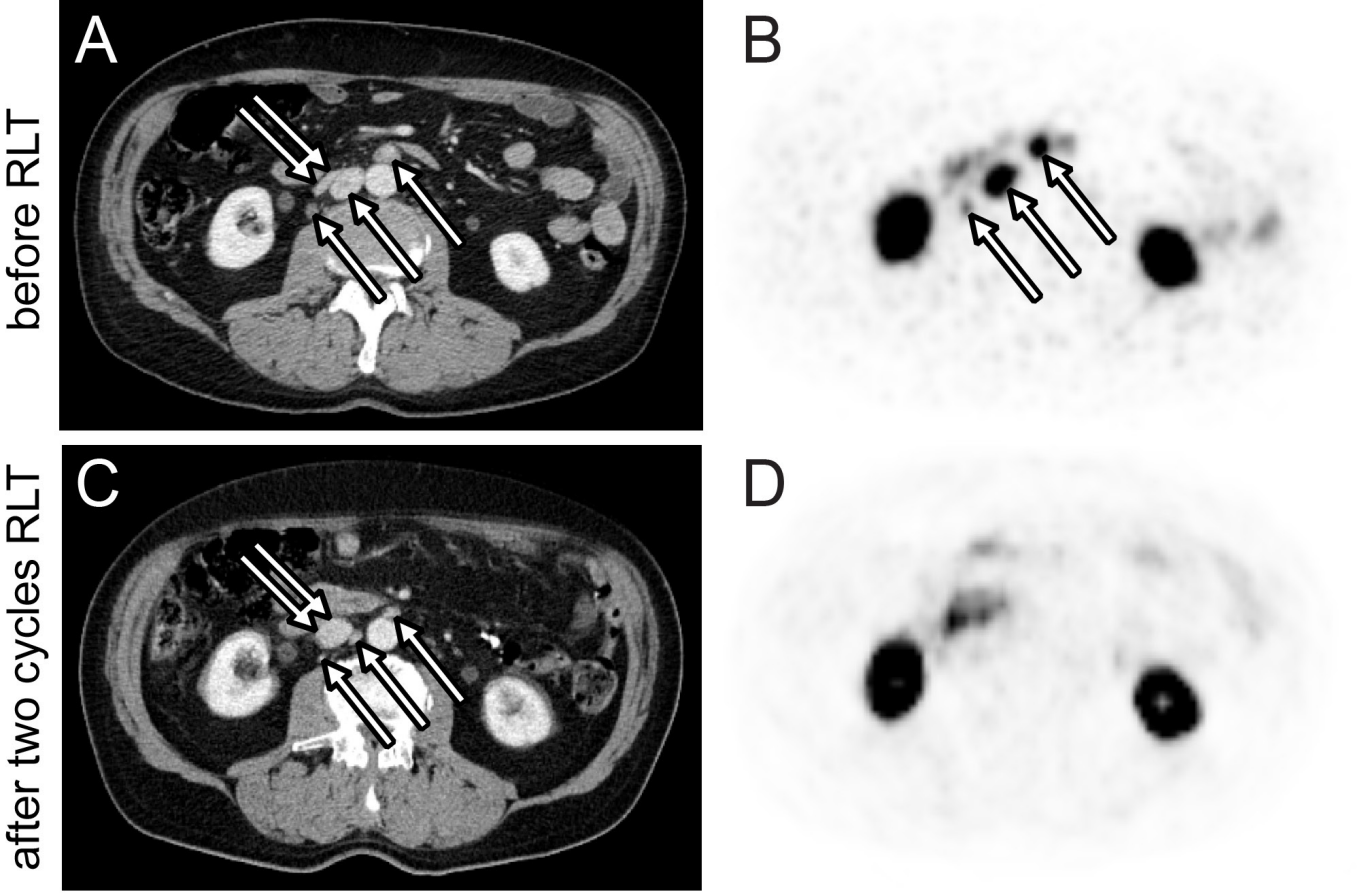
Response after two cycles of 6.0 GBq ^**177**^Lu-PSMA-617 RLT Axial ^68^Ga-PSMA PET **B.** and **D.** and CT **A.** and **C.** images of the abdomen before (A and B) and after (C and D) two RLT cycles. Lymph node metastases demonstrate a >30% baseline to follow-up decrease in short axis diameter (A and C, arrows) and SUV_max_ (B, arrows). Compression of the inferior vena cava by lymph node metastases at baseline (A, double arrow) was resolved at follow-up (C, double arrow).

**Figure 2:**
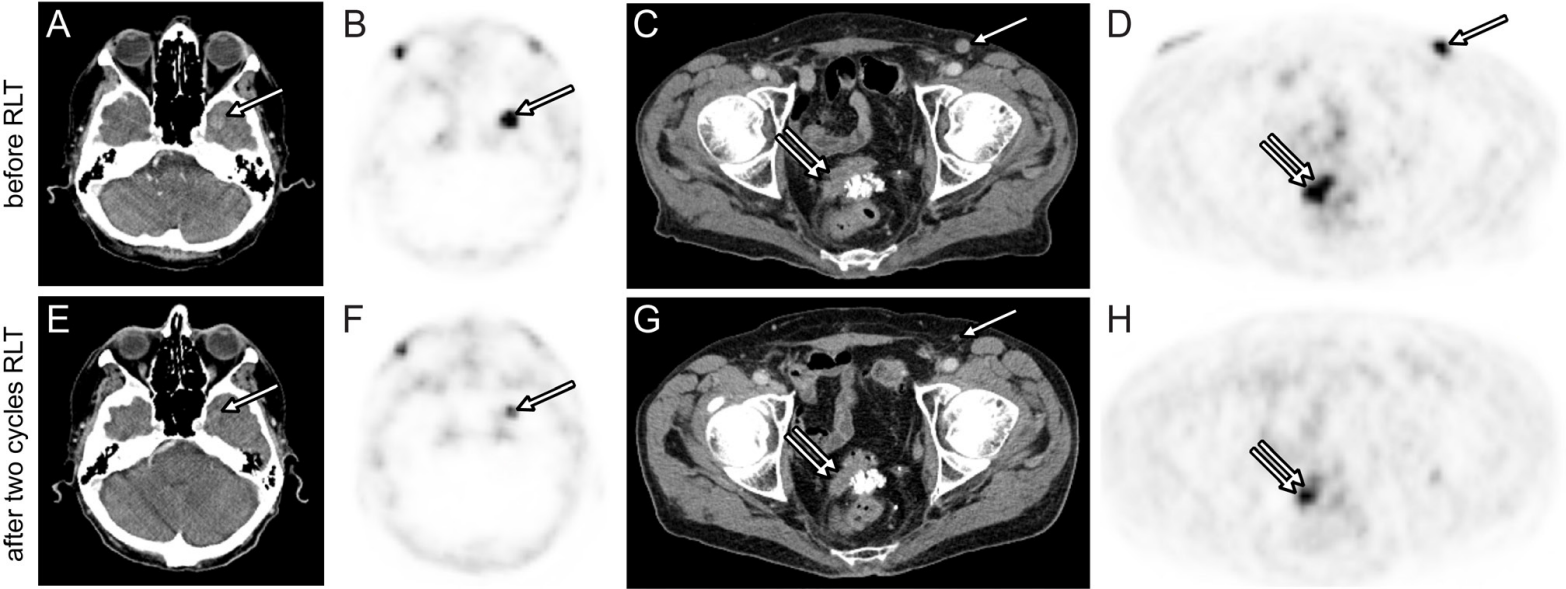
Response after two cycles of 6.0 GBq ^**177**^Lu-PSMA-617 RLT Axial ^68^Ga-PSMA PET **B.**, **D.**, **F.**, **H.** and CT **A.**, **C.**, **E.**, **G.** images of the base of the skull and the pelvis before (A, B, C, D) and after (E, F, G, H) two RLT cycles. Brain metastasis (A, B, E, F, arrow) and local recurrence (C, D, G, H, double arrow) demonstrate a >30% baseline to follow-up decrease in largest diameter and SUV_max_. Inguinal lymph node metastasis (C, D, G, arrow) shows complete response on follow-up PET/CT.

Response after one cycle and two cycles of RLT is given separately in Table 4. Ten (67%) patients had measurable target lesions by RECIST1.1. According to RECIST1.1, 4 (27%) patients had PR, 6 (40%) patients had SD, and 5 (33%) patients had PD. Images from two patients with partial response by CT criteria are shown in Figures 1 and 2. Each 7 (47%) patients had biochemical partial response by PSA level after the first and second cycle, respectively. Bone pain was completely resolved in 3 (20%) and responded well in 4 (27%) symptomatic patients after two RLT cycles. One and 3 (20%) patients had increase in pain intensity after the first and second RLT cycle, respectively. QoL was improved in 8 (53%) patients and 3 (20%) patients had a 30% or more increase in QoL score after the second cycle.

**Table 4:** Response after two cycles

*n*=15		Objective response			Patient reported outcomes	
		RECIST*	PSA		Pain	QoL
After one cycle						
CR		-	0		1 (7%)	-
PR		-	7 (47%)**		6 (40%)	-
SD		-	5 (33%)		2 (13%)	-
PD		-	3 (20%)		1 (7%)	-
After two cycles						
CR		0	0		3 (20%)	0
PR		4 (27%)	7 (47%)**		4 (27%)	3 (20%)
SD		6 (40%)	5 (33%)		0	11 (73%)
PD		5 (33%)	3 (20%)		3 (20%)	1 (7%)

* primary outcome,

** ≥30% decline in PSA; Abbreviations: RECIST, Response Evaluation Criteria in Solid Tumors; PSA, Prostate-Specific Antigen; QoL, quality of life; SD, stable disease; PR, partial response; PD, progressive disease; CR, complete response.

**Figure 3:**
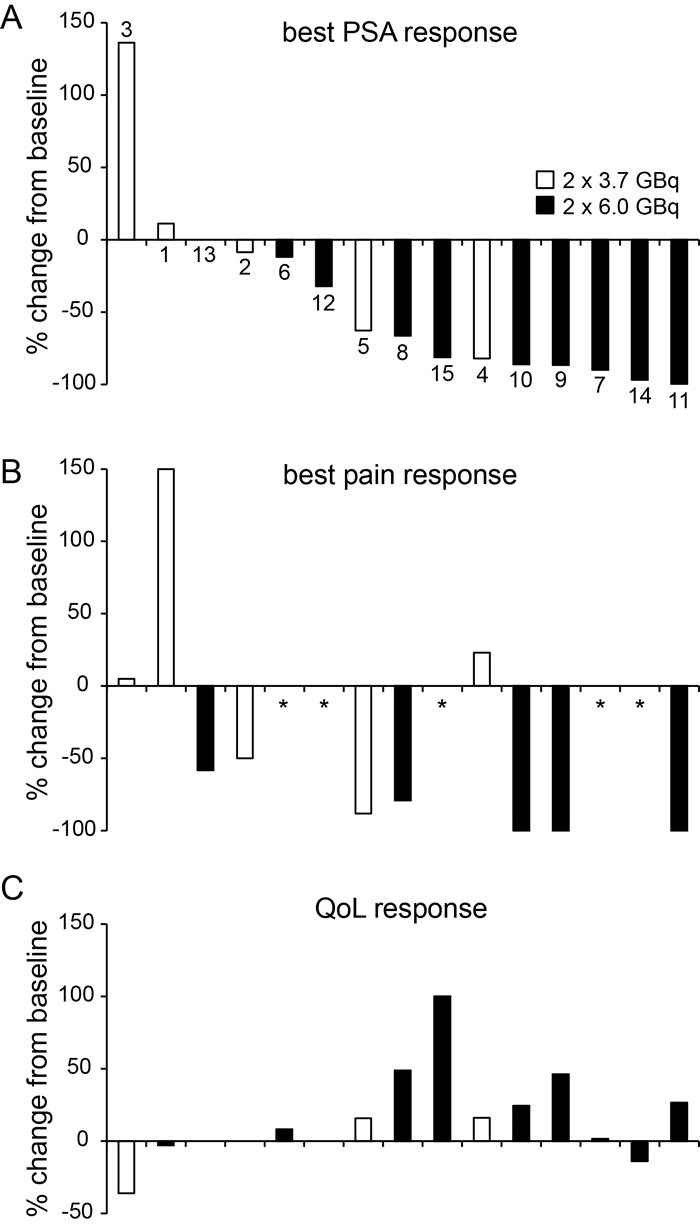
Best response after 30 cycles of ^**177**^Lu-PSMA-617 RLT in 15 patients Individual response by lowest PSA **A.**, lowest pain **B.** and quality of life (QoL) level **C.** from start until follow-up after the second RLT cycle is shown. Individual results were arranged by best PSA response and separated by regimen (white bars: 2×3.7 GBq; black bars: 2×6.0 GBq). Patient number is given in (A). Bars indicate baseline to follow-up change in percent. *no baseline pain noted.

Best response observed for PSA, pain score and final response by QoL score are shown for each patient in Figure 3. Nine (60%) patients had a PSA decline of 50% or more during RLT. Response by baseline to follow-up change in AP level or ^68^Ga-PSMA uptake are given in Supplemental Table 1.

## DISCUSSION

Dosimetry, safety and efficacy of ^177^Lu-PSMA-617 RLT were analyzed in 15 patients after a total of 30 applications. Treatment response was determined by RECIST1.1 and a range of secondary endpoints, including change in PSA and patient reported outcomes (pain, quality of life). In general, we find that ^177^Lu-PSMA-617 RLT was tolerated well for both activity regimens, i.e. 3.7 or 6.0 GBq per cycle. Dose limits for critical organs (kidney, 23 Gy [7]; salivary glands, 26-50 Gy [8-9]) were not exceeded in any patient. Eight to ten weeks after the second application, tumor extent on CT had stabilized or showed significant reduction in 10 patients corresponding to a disease control rate of 67%. Approximately half of patients experienced pain relief and improvement in QoL after RLT.

Endoradiotherapy using chelator-bound ligands present several advantages over conventional therapy for metastatic prostate cancer. Whereas the ^68^Ga-labelled compound provides pre-therapeutic staging and target expression levels to PET, the corresponding ^177^Lu-labelled compound enables tumor irradiation through focal beta emission, while also enabling imaging and dosimetry through partial gamma emission. High efficacy and safety of ^177^Lu-based RLT was recently proven in a phase III study of patients with advanced-stage mid-gut neuroendocrine tumors (NET). In that study, somatostatin analogue combination therapy with octreotide plus ^177^Lu-DOTATATE versus octreotide alone demonstrated approximately 80% risk reduction for NET progression, with few serious adverse events [10]. Chelating ligands with high affinity to PSMA have been developed for imaging (^68^Ga-HBED-PSMA) and RLT (^177^Lu-PSMA-617) in order to translate this concept into prostate cancer patients [5, 11]. We proved favourable dose distribution, and determined safe initial activity for ^177^Lu-PSMA-617 RLT (i.e. 6.0 GBq) in an earlier dosimetry trial [6]. This cohort was now expanded by ten patients completing 20 cycles of 6.0 GBq RLT so as to analyze better the clinical safety and efficacy of this regimen. In this expanded cohort, mean tumor-to-kidney dose ratio was higher than the ratio reported for ^177^Lu-DOTATATE, which is the clinical standard for RLT in NET patients [12]. More accurate dosimetry using quantitative SPECT reveals an approximately 20 to 30% lower radiation dose to salivary glands than previously estimated by planar scintigraphy [6, 13-14]. Accordingly, the critical dose limits bringing loss of salivary function were not exceeded in any patient. Indeed, serious adverse events for kidney or salivary gland function were not recorded, indicating an excellent safety profile of ^177^Lu-PSMA-617 RLT. Consistent with dosimetry and previous clinical findings [14-17] the rate of hematologic adverse events was low and few hematologic conditions were transient and not life threatening.

Several trials have earlier shown effective reduction in tumor burden and serum PSA levels after administration of ^177^Lu-PSMA-617 [14-20]. About one third to half of our fifteen patients showed significant improvement after two cycles based on CT size and PSA level. Most remarkably, one patient with left temporal brain metastasis refractory to external beam radiotherapy demonstrated partial response after RLT (Figure 2). PSA decline of 50% and more during RLT was recorded in nine patients, corresponding to a best PSA response rate of 60%. This rate is consistent with prior reports from Ahmadzadehfar et al [15], Kratochwil et al [14] and with findings for other radio-labelled PSMA ligands [21-22]. Rahbar et al and Heck et al demonstrate similar rates for PSA decline after one and two RLT cycles respectively [16, 23]. When compared to findings from a recent study on mCRPC patients, the best PSA response was roughly similar to that for Abiraterone treatment and higher than for Enzalutamide treatment [24]. Notably, all patients with RECIST partial response and 85% of patients with ≥30% final PSA decline underwent the 6.0 GBq RLT regimen. However, single biomarker tests for treatment response are unreliable in end-stage prostate cancer patients [25]. Accuracy of CT response is limited by tumor dissemination and the frequent presence of non-target bone lesions. Change in PSA or AP levels show only modest correlation with clinical benefit [26]. We thus chose to systematically record patient-reported outcomes using established instruments (Brief Pain Inventory, FACT-P) in order to understand better the clinical response to RLT. Patient reported outcome was favourable in those of our patients with decreasing PSA level during ^177^Lu-PSMA-617 RLT: Seven (47%) patients experienced significant pain relief and 8 (53%) reported increased QoL score after the second cycle.

By including broad range of objective and patient-reported biomarker endpoints, our study provides a detailed analysis of the efficacy of ^177^Lu-PSMA-617 RLT. Safety was thoroughly documented by dosimetry and frequent follow-up visits for all patients. The present study however has limitations. Although patient inclusion criteria and treatment protocols were based upon pre-defined institutional standards, our retrospective data analysis may have increased the risk for underreporting of adverse events. Furthermore, our inclusion criteria resulted in a small patient cohort, which was nonetheless representative of mCRPC patients seen at our clinic. Furthermore 10 to 20% error for radiation dose calculation might be caused by omitting late SPECT acquisition as previously estimated for planar scintigraphy [27]. Overall results are promising however must be confirmed in a randomized trial with longer follow-up period in order to monitor for radiation induced, delayed events.

In summary, two cycles of ^177^Lu-PSMA-617 RLT were safe and resulted in promising response rates for objective and patient-reported outcomes in fifteen patients with metastatic castration-resistant prostate cancer. Based on dosimetry more than three cycles of 6.0 GBq RLT can be performed with acceptable safety margin. Our preliminary data encourage future investigations of ^177^Lu-PSMA-617 RLT in a multicentre study of prospective design.

## MATERIALS AND METHODS

### Patients

Between September 2014 and May 2016 thirty cycles of ^177^Lu-PSMA-617 RLT were applied in 15 consecutive patients. The initial five patients received 2 × 3.7 GBq (*n* = 10 cycles). Those patients had also been included in our previous report on ^177^Lu-PSMA-617 dosimetry [6]. The following five patients received a higher dose (2 x 6.0 GBq, *n* = 20 cycles) based on our previous recommendation [6]. RLT was offered in accordance with the current Declaration of Helsinki, paragraph 37 “Unproven Interventions in Clinical Practice” and in accordance with German compassionate use regulations. Indication was confirmed by both a nuclear medicine physician and a urologist or oncologist. Patients meeting the following criteria, consistent with the German Society of Nuclear Medicine consensus panel recommendation [28], were considered for RLT: (a) prostate cancer proven by histopathology, (b) unresectable metastases, (c) castration resistant disease, (d) completed on-label treatment options for castration-resistant disease or unfit for such treatments, (e) objective disease progression by PSA level and imaging, (f) PSMA-avid lesions on pre-therapeutic ^68^Ga-HBED-PSMA positron emission tomography/computed tomography (PET/CT), (g) white blood cell count (WBC) >3000/μl and platelet count >75000/μl, (h) creatinine <2-fold the upper limit of normal (ULN), (i) AST and ALT <5-fold ULN, and (j) no myelosuppressive therapy within six weeks prior to RLT. All patients gave written informed consent to undergo RLT with subsequent follow-up. Analyses presented in this study were performed retrospectively on anonymized patient data. The study protocol was approved by the local ethics committee and all subjects had provided prior written informed consent.

### 177Lu-PSMA-617 RLT

DOTA-PSMA-617 precursor was obtained from ABX GmbH (Radeberg, Germany). In brief, DOTA-PSMA-617 was dissolved in 1.5 ml of acetate buffer (pH 4.8) containing 10 mg/ml dihydroxybenzoic acid. The solution was added to no-carrier-added ^177^LuCl_3_ in 0.04M HCl obtained from ITG GmbH (Garching, Germany), and heated for 30 min at 90 to 100°C. The amount of precursor was 20 μg/GBq ^177^Lu. Radiochemical yield of ^177^Lu-PSMA-617 was greater than 95% and radiochemical purities greater than 99% were achieved in each final PBS-buffered preparation. Each treatment was performed during a four days stay at the Nuclear Medicine ward. ^177^Lu-PSMA-617 was given by slow i.v. application over twenty minutes. Patients received 50 mg prednisolone and 1L of 0.9% NaCl i.v. or equivalent amounts of p.o. fluids daily until discharge. In order to reduce blood flow and radioligand uptake in parotid and submandibular glands, ice packs were applied locally from ten min prior to ^177^Lu-PSMA-617 administration, and continued for six hours thereafter.

### Safety and dosimetry

A flow diagram for treatment and follow-up is given in Supplemental Figure S1. All patients were monitored daily as in-patients for sub-acute toxicity, with physical examination and routine bloodwork for electrolytes, hematology, liver and kidney function. All patients had two follow-up examinations at four to five and eight to ten weeks after each RLT cycle, including routine bloodwork and one ^99m^Tc-MAG3 renal scintigraphy to monitor latent toxicity. The observation period ended on January 31^st^ 2016. Adverse events for total WBC were graded by CTCAE 3.0 [29]. Adverse events for tubular extraction rate (TER) were defined as follows: grade 1, 67-100% lower limit of normal (LLN); grade 2, 33-66% LLN; grade 3, 0-32% LLN. The National Cancer Institute's Common Terminology Criteria for Adverse Events (CTCAE) Version 4.0 was used for all other toxicity [30].

Whole body and organ dosimetry was obtained at each cycle (*n* = 30) as described previously [6]. Additional quantitative SPECT/CT of the head was performed approximately 24, 48 and 72h after each application of 6.0 GBq ^177^Lu-PSMA-617 in 10 patients (*n* = 20) to accurately calculate radiation dose to salivary glands, the organ with highest absorbed radiation dose in our initial study [6].

### Response to RLT and statistical analysis

^68^Ga-HBED-PSMA PET/CT had been performed one to two weeks before the first RTL cycle, and again at eight to ten weeks following the second RLT cycle, as reported previously [31]. Primary endpoint for efficacy was response as determined by RECIST 1.1. Secondary endpoints were change in PSA level, pain intensity score, and QoL score. PSA and, in case of bone metastases, AP blood levels were measured at baseline and each subsequent visit. Progressive disease (PD) was defined as 30% or more increase, partial response (PR) as 30% or more decrease in final PSA/AP levels relative to baseline. Symptomatic patients reported all four pain severity items of the Brief Pain Inventory (BPI) [32] at baseline and again at eight to ten weeks after each cycle. In addition, any change in pain medication was documented. Here, PD was defined as a 30% or greater increase in pain score or increase in pain medication, whereas PR was defined as corresponding 30% or greater decrease or decrease in pain medication. CR was defined as pain score 0 on final follow-up. Patients reported QoL based on Functional Assessment of Cancer Therapy - Prostate questionnaire (FACT-P) [33] at baseline and at eight to ten weeks after the second cycle, with PD and PR defined as changes of at least 30% relative to baseline QoL score. Stable disease (SD) was each defined as non-PD/PR. Results are presented as total number (percent), mean ± standard deviation (SD), or median (range). Change in baseline to follow-up SUV_max_ was determined as exploratory data for up to three lesions with highest SUV_max_ on baseline ^68^Ga-HBED-PSMA PET. Details for AP and PET response are given in the Supplemental Material.

## SUPPLEMENTARY MATERIALS FIGURES AND TABLES


