# Supplementary material for: Preliminary experience with dosimetry, response and patient reported outcome after 177Lu-PSMA-617 therapy for metastatic castration-resistant prostate cancer
Source: Oncotarget. 2016 Sep 24;8(2):3581–90. doi: 10.18632/oncotarget.12240 (PMC5356905; doi:10.18632/oncotarget.12240)
Supplement: Supplementary file 1 [file oncotarget-08-3581-s001.pdf]

Preliminary experience with dosimetry, response and patient reported outcome after ^{177}Lu-PSMA-617 therapy for metastatic castration-resistant prostate cancer

Supplementary Material

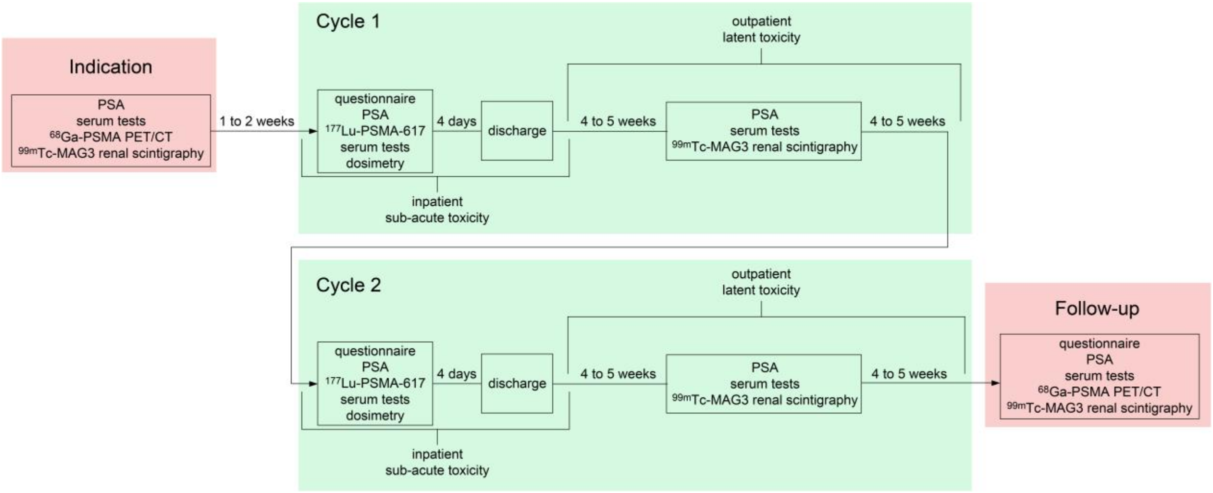

Supplemental Figure S1. Flow chart for ^{177}Lu-PSMA-617 RLT and follow-up.

Supplemental Table 1. Response eight to ten weeks after the second cycle according to ^{68}Ga-PSMA PET and AP level

Category	PET	AP
PR	11 (73%)	2 (13%)
SD	3 (20%)	7 (47%)
PD	1 (7%)	5 (33%)

Abbreviations: SD, stable disease; PR, partial response; PD, progressive disease.

Response to RLT - Details

PET response was determined by baseline to follow-up change in sum of SUV_{max} of all lesions (to a maximum of three lesions per organ). Based on PERCIST 1.0 [1], progressive disease (PD) was defined as 30% or greater increase in summed SUV_{max}, whereas partial response (PR) was defined as 30% or greater decrease in SUV_{max}.

References

1. Wahl RL, Jacene H, Kasamon Y and Lodge MA. From RECIST to PERCIST: Evolving Considerations for PET response criteria in solid tumors. J Nucl Med. 2009; 50 Suppl 1:122S-150S.
